# Hematospermia in a Transgender Woman with Evidence for Endometrial Tissue in the Prostate

**DOI:** 10.1016/j.aace.2024.01.006

**Published:** 2024-01-24

**Authors:** Janet Coleman-Belin, Uchechukwu O. Amakiri, Fang-Ming Deng, Deepthi Hoskoppal, Joshua D. Safer, Tamar Reisman

**Affiliations:** 1 Icahn School of Medicine at Mount Sinai, New York, New York; 2 Mount Sinai Center for Transgender Medicine and Surgery, New York, New York; 3 Department of Pathology and Urology, New York University Langone School of Medicine, New York, New York; 4 Department of Pathology, New York University Langone School of Medicine, New York, New York; 5 Division of Endocrinology, Department of Medicine, Mount Sinai Morningside and Mount Sinai West, Icahn School of Medicine at Mount Sinai, New York, New York

**Keywords:** hematospermia, gender, transgender and gender diverse (TGD), gender-affirming hormone therapy (GAHT), endometriosis, prostate

## Abstract

**Background/Objective:**

The frequency of hematospermia in transgender women is unknown. This report aimed to describe the development of hematospermia in a transgender woman.

**Case Report:**

A 35-year-old transgender woman treated with estradiol valerate and leuprolide presented with painless rust-tinged ejaculate, urethral bleeding after ejaculation, and intermittent hematuria. Her medical history included gastroesophageal reflux disease, internal hemorrhoids, and attention deficit hyperactivity disorder with negative tobacco smoking and urologic history. Additional medications included emtricitabine-tenofovir disoproxil fumarate and fexofenadine. Physical examination did not reveal constitutional or genitourinary abnormalities. Urinalysis and culture disclosed rare white blood cells with gram-variable bacilli. The chlamydia, gonorrhea, and human immunodeficiency virus test results were negative. Abdominal computed tomography did not reveal bladder or prostate cancer, calcifications, inflammation, or cysts. She continued to have symptoms after this initial workup. One year after the initial symptom onset, transrectal ultrasound disclosed a 1.7-cm midline posterior prostatic cyst with hemorrhagic products, later revealed by magnetic resonance imaging as communicating with the left seminal vesicle. Two ultrasound-guided transperineal biopsy samples revealed benign prostatic tissue with a small focus of Müllerian or endometrial-type tissue, evidenced by immunopositivity for paired-box gene 8 and estrogen receptor in epithelium and cluster of differentiation 10 immunopositivity in stroma. After medical consultation, the patient underwent prostatic cyst aspiration, resection of the transurethral ejaculatory ducts, and orchiectomy. She did not experience any complications after these procedures.

**Discussion:**

The etiology of hematospermia may be idiopathic, iatrogenic, anatomic, or pathologic.

**Conclusion:**

Occult endometriosis or ectopic Müllerian epithelial tissue growth may occur in transgender women taking feminizing gender-affirming hormone therapy.

**Highlights**

• Endometrial tissue is a rare cause of hematospermia
• Endometrial tissue growth may be linked to feminizing hormone therapy
• This case highlights important of multidisciplinary collaboration in transgender care

**Clinical Relevance**

This case report underscores the need for heightened clinical awareness of potential atypical presentations in transgender women undergoing hormone therapy. By shedding light on the possibility of endometrial tissue growth, health care providers can enhance diagnostic accuracy, improve patient care, and ensure timely intervention in this specific population.

## Introduction

Hematospermia—defined as the macroscopic presence of bloody or blood-tinged seminal ejaculate—is rare and usually self-limited. Although often concerning to patients, hematospermia is typically benign.^1^, ^2^, ^3^ Grossly visible blood in semen may be idiopathic or iatrogenic from urologic surgery or due to inflammation such as with sexually transmitted infections, *Mycobacterium tuberculosis* disease, systemic hypertension, hemorrhage, seminal vesicle stones, prostatic/ejaculatory duct cysts, calcifications, Müllerian duct remnants, and prostate cancer.^1^^,^^4^^,^^5^ Patients aged >40 years with factors considered to be high risk, including recurrent symptoms, hematuria, and a history of prostate cancer, are referred to urology.^6^ A history of urologic surgery is associated with an increased incidence of hematospermia The incidence of hematospermia in the general population is unknown.^3^ Furthermore, neither the frequency nor presentation of hematospermia in transgender women on gender-affirming hormone therapy (GAHT) is currently documented in existing literature. This report provides a case of a transgender woman who presented with hematospermia and describes the potential development of the condition in this individual patient.

## Case Report

A 35-year-old transgender woman presented with painless rust-tinged ejaculate and intermittent urethral bleeding after ejaculation, along with 1 year of intermittent hematuria. She had a medical history of gastroesophageal reflux disease, internal hemorrhoids, and attention deficit hyperactivity disorder. Her medications at the time were estradiol valerate and leuprolide for 1 year, emtricitabine-tenofovir disoproxil fumarate for human immunodeficiency virus prevention, and fexofenadine for seasonal allergies. One and a half years before presentation, she had formerly taken bicalutamide and progesterone for 2 months but discontinued these because she felt fatigued. On physical examination, she was alert and oriented in no apparent distress. Her blood pressure, temperature, respiratory rate, oxygen saturation level, and body mass index were 106/61 mm Hg, 98°F, 16 breaths/minute, 88%, and 26.3 kg/m^2^, respectively. She was normocephalic with moist and clear mucous membranes. Cardiovascular examination revealed a regular rate and rhythm with no murmurs, rubs, or gallops. The lungs were clear bilaterally to auscultation and percussion with no cough, sputum, or consolidation. She had normoactive bowel sounds, costovertebral tenderness, and focal neurologic deficits. Her mood, affect, and thought content were normal. She had no urologic history and was a lifetime nonsmoker.

To rule out certain and more common reasons for her presentation, the patient underwent varying evaluations. Urinalysis and culture revealed rare white blood cells with gram-variable bacilli. The chlamydia, gonorrhea, and human immunodeficiency virus test results were negative. Abdomen and pelvic computed tomography revealed a normal prostate, no renal or ureteric abnormalities bilaterally, and no evidence of nephrolithiasis or a recently passed stone. Cystoscopy identified a normal urethra, bladder neck, and inner bladder with no occlusions, tumors, erythema, inflammation, bladder stones, or diverticula. She continued to have hematospermia and hematuria after this initial workup.

**Fig. 1:**
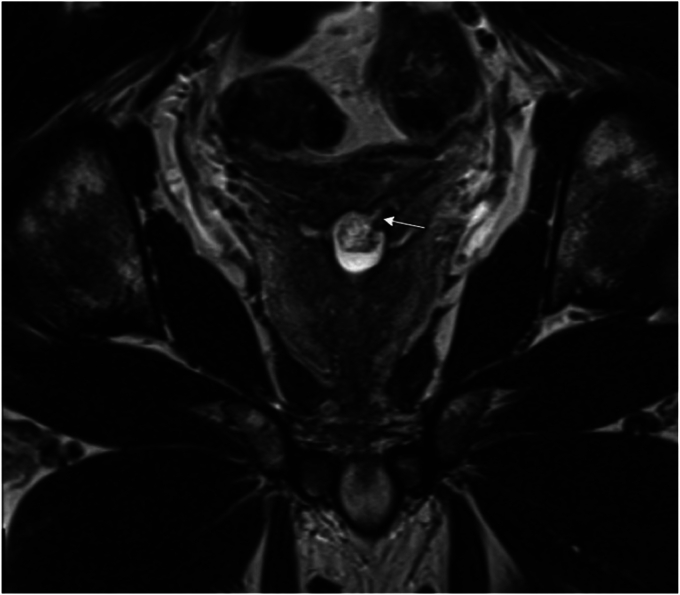
In a 35-year-old transgender woman presenting with hematospermia, magnetic resonance imaging revealed a 1.7-cm posterior prostatic cyst communicating with the left seminal vesicle.

One year after the initial symptom onset, transrectal ultrasound revealed a normal-sized prostate and 1.7-cm midline posterior prostatic cyst with hemorrhagic products. Magnetic resonance imaging of the prostrate noted a prostate size of 3.9 × 2.6 × 4.1 cm (for an overall volume of 21.6 mL) with a 1.7 × 1.4 × 1.7-cm midline posterior prostatic cyst communicating to the left seminal vesicle and no findings suspicious for a prostate cancer (Fig. 1).

**Fig. 2:**
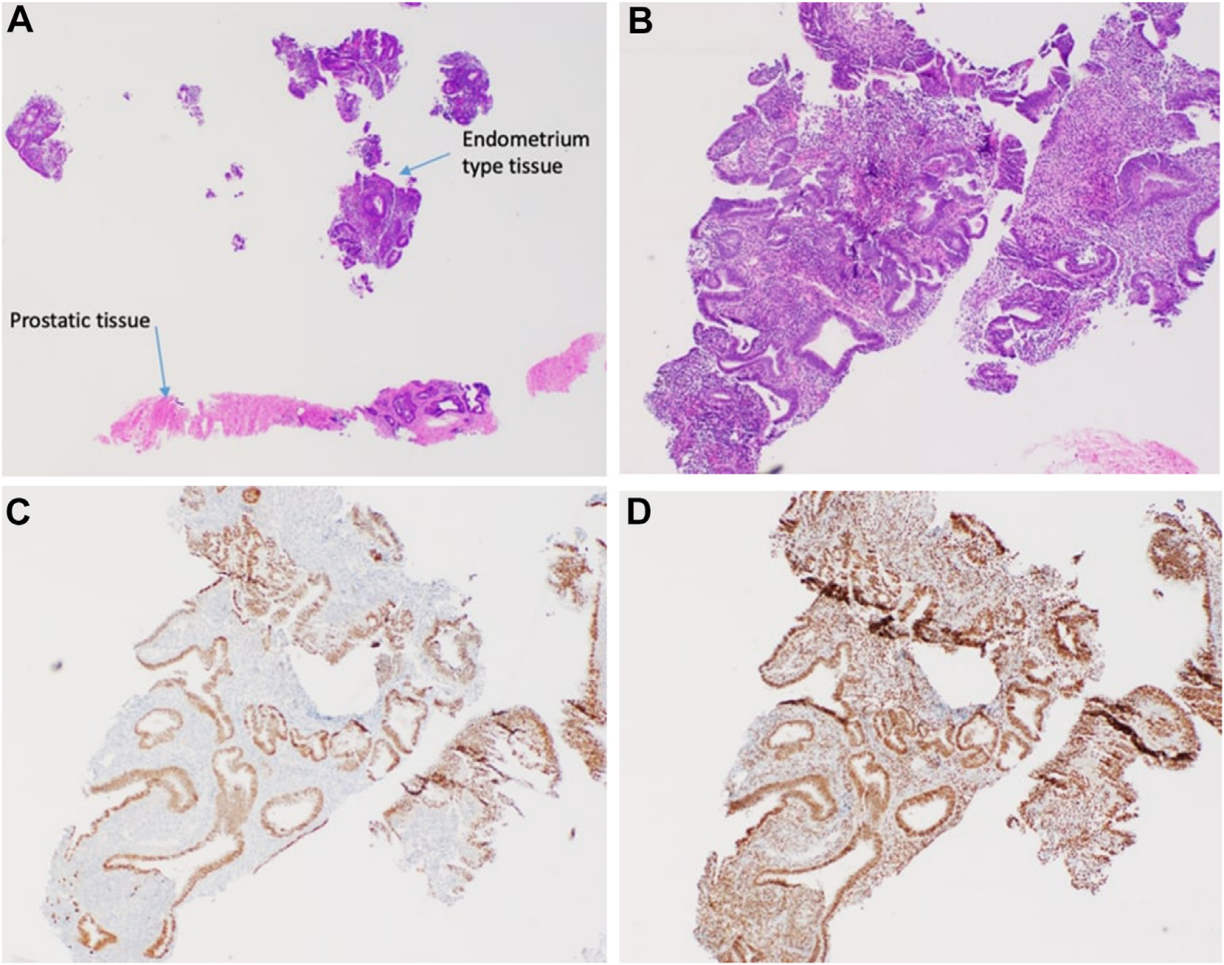
Pathology of 2 ultrasound-guided transperineal biopsy samples of the patient’s posterior prostatic cyst with hemorrhagic products. Histopathology images were labeled as hematoxylin-eosin stains with magnifications of ×20 (*A*) and ×40 (*B*) of endometrium-type tissue. Immunostains specific for Müllerian or endometrial-type tissue were paired-box gene 8 immunostain (*C*) and estrogen receptor immunostain (*D*).

Two ultrasound-guided transperineal biopsy samples revealed benign prostatic tissue with a small focus of Müllerian or endometrial-type tissue, supported by immunopositivity for paired-box gene 8 and estrogen receptor immunostains in epithelium along with a cluster of differentiation 10 immunostain positivity in stroma. The pathology report noted that these findings could indicate either endometriosis or a Müllerian cyst (Fig. 2 *A* through *D*).

The patient underwent prostatic cyst aspiration, resection of the transurethral ejaculatory ducts, and orchiectomy. The histopathology on the aspiration was similar to the previous biopsy. Hematospermia symptoms ceased upon aspiration of the prostatic cyst with endometrial-type tissue. Surgical pathology of the orchiectomy specimen revealed bilateral testes with atrophy and seminiferous tubules that exhibited arrest in spermatogenesis without any spermatids. Additionally, the left testis had areas with marked tubular hyalinization. The patient did not experience any complications after these procedures. Six months after transperineal biopsy, she was medically cleared for vaginoplasty.

## Discussion

Endometriosis is microscopically described as the presence of endometrial-like epithelium, glands, and stroma outside of the endometrium and myometrium and can be of Müllerian or non-Müllerian embryologic origin.^7^ Paired-box gene 8 immunostain is sensitive and specific for any Müllerian-derived epithelia, and estrogen receptor immunostain is commonly expressed in endometrial epithelium to allow for cell-specific responses (trophic and mitotic) during the proliferative phase of the menstrual cycle.^8^ Cluster of differentiation 10 immunostain is positive in endometrial stroma but not in ovarian stroma.^9^ Together, the immunostaining pattern is consistent with Müllerian-derived endometrial tissue with stroma. Considering the clinical history and a well-demarcated cyst on imaging, this may suggest a Müllerian cyst with endometrial differentiation (both glands and stroma) or endometriosis. It is difficult to differentiate between endometriosis and a Müllerian cyst based on histology and immunohistochemistry alone. The connection between the cyst and left seminal vesicle as noted on the magnetic resonance imaging finding could explain the hematospermia.

Müllerian tissue, and more specifically, the Müllerian ducts comprise structures involved in the development of the internal genital portions of the female reproductive system. Although the Müllerian ducts are initially present in all individuals, when not acted upon by the Y chromosome–associated anti-Müllerian hormone (AMH)—as is the case in cisgender women—the Müllerian ducts develop into the fallopian tubes, uterus, cervix, and proximal third of the vagina. Endometrial tissue is generally a derivative of Müllerian stem cells and does not typically arise in cisgender men born with internal and external male anatomy because they express AMH.^10^^,^^11^ Albeit rare, endometriosis in cisgender men has previously been reported in the literature and presents primarily with abdominal pain.^6^^,^^12^, ^13^, ^14^ The proposed mechanisms for this presentation in these cisgender men included sequelae of extended estrogen exposure or inflammation.^6^^,^^12^, ^13^, ^14^

Because the patient presented with hematospermia and high-risk factor of concurrent hematuria, she underwent extensive evaluation because the condition is associated with multiple etiologies, including inflammation, infection, obstruction, systemic disease, trauma, vascular abnormalities, and rarely prostate cancer.^10^^,^^11^ The results of the patient’s testing were within normal limits, leading to exploration of other explanations of her hematospermia, including potential endometriosis (microscopic and macroscropic) or ectopic Müllerian epithelial tissue.^7^

The induction theory of endometriosis hypothesizes that ectopic endometrial tissue develops from pathologic changes to embryonic tissue.^15^ Cisgender men could theoretically have residual primordial tissue if the Müllerian ducts imperfectly disintegrated when exposed to AMH.^6^^,^^12^^,^^15^ The prostatic utricle, an epithelium-lined remnant of the Müllerian duct in males, is a small sac-like indentation on the posterior prostatic urethra. Müllerian cells susceptible to differentiating could theoretically persist in this region.^6^^,^^12^ Exposure to estrogen, inflammation, or other processes could then induce cell differentiation into endometrial tissue. This theory is consistent with the posterior prostatic location of the endometriosis in the present case, as well as in most case reports of endometriosis in cisgender males.^6^

The etiology of this condition is also hypothesized to include liver disease or chronic postoperative inflammation.^6^^,^^13^^,^^14^^,^^16^ Insufficient immune system response may be at fault^6^^,^^15^ and has been posited as another theory of ectopic endometrial growth in cisgender women.^15^

In cisgender women, endometriosis management is usually symptom directed. The first-line treatments are acetaminophen, nonsteroidal anti-inflammatory drugs, and combined hormonal contraceptives, whereas the second-line treatments for endometriosis are gonadotropin-releasing hormone agonists. Refractory cases not responsive to subsequent specialized medications are managed with surgical excision.^17^ Malignant transformation is uncommon, and approximately only 1% of cisgender women with endometriosis develop endometriosis-associated ovarian carcinoma.^18^ As such, a history of ectopic endometrial tissue is associated with a higher relative risk of developing endometriosis-associated ovarian carcinoma.^18^ It is of interest to determine whether the rates of malignant transformation differ in transgender women on feminizing GAHT.

To the best of our knowledge, this case report may be the first documentation of endometrial tissue in a transgender woman. There may be more endometrial epithelium and stroma in transgender women on GAHT than previously understood. In addition, this case report can serve as evidence against retrograde transport of menstrual tissue as the cause of endometriosis in cisgender women^6^^,^^15^ and suggests that the induction theory of endometriosis is more likely.

## Disclosure

The authors have no conflicts of interest to disclose.
